# Folate-modified liposomes mediate the co-delivery of cisplatin with miR-219a-5p for the targeted treatment of cisplatin-resistant lung cancer

**DOI:** 10.1186/s12890-024-02938-6

**Published:** 2024-04-01

**Authors:** Yuanlin Wu, Jiandong Zhang, Junjun Zhao, Bin Wang

**Affiliations:** Department of Thoracic Surgery, Shaoxing People’s Hospital, No.568 Zhongxing North Road, 312000 Shaoxing, Zhejiang China

**Keywords:** DDP-resistance, FA, miR-219a-5p, NSCLC, Nano-drug delivery system

## Abstract

Cisplatin (DDP) resistance, often leading to first-line chemotherapy failure in non-small cell lung cancer (NSCLC), poses a significant challenge. MiR-219a-5p has been reported to enhance the sensitivity of human NSCLC to DDP. However, free miR-219a-5p is prone to degradation by nucleases in the bloodstream, rendering it unstable. In light of this, our study developed an efficient nanodrug delivery system that achieved targeted delivery of DDP and miR-219a-5p by modifying liposomes with folate (FA). Based on the results of material characterization, we successfully constructed a well-dispersed and uniformly sized (approximately 135.8 nm) Lipo@DDP@miR-219a-5p@FA nanodrug. Agarose gel electrophoresis experiments demonstrated that Lipo@DDP@miR-219a-5p@FA exhibited good stability in serum, effectively protecting miR-219a-5p from degradation. Immunofluorescence and flow cytometry experiments revealed that, due to FA modification, Lipo@DDP@miR-219a-5p@FA could specifically bind to FA receptors on the surface of tumor cells (A549), thus enhancing drug internalization efficiency. Safety evaluations conducted in vitro demonstrated that Lipo@DDP@miR-219a-5p@FA exhibited no significant toxicity to non-cancer cells (BEAS-2B) and displayed excellent blood compatibility. Cellular functional experiments, apoptosis assays, and western blot demonstrated that Lipo@DDP@miR-219a-5p@FA effectively reversed DDP resistance in A549 cells, inhibited cell proliferation and migration, and further promoted apoptosis. In summary, the Lipo@DDP@miR-219a-5p@FA nanodrug, through specific targeting of cancer cells and reducing their resistance to DDP, significantly enhanced the anti-NSCLC effects of DDP in vitro, providing a promising therapeutic option for the clinical treatment of NSCLC.

**Supplementary Information:**

The online version contains supplementary material available at 10.1186/s12890-024-02938-6.

## Introduction

Lung cancer is the second most common cancer worldwide, accounting for 11.4% of all cancer cases, and is a primary contributor to cancer-related deaths [1–3]. Non-small cell lung cancer (NSCLC) comprises a large proportion (85%) of all lung cancer patients and is a serious threat to human life. Platinum-based combination chemotherapy is currently the standard first-line treatment for advanced NSCLC patients. Cisplatin (DDP), a first-generation platinum-based drug, is one of the most commonly used drugs in NSCLC chemotherapy [4, 5]. However, prolonged clinical use of DDP leads to a decrease in tumor cell sensitivity to chemotherapy, which is a major cause of chemotherapy failure. Advances in molecular biology have enabled people to gain a profound comprehension of drug resistance in tumor cells [6–8]. Examining the genes responsible for drug resistance in NSCLC is thus a crucial step toward improving the diagnosis and cure rate of patients.

MicroRNAs (miRNAs) are short, non-coding RNAs, typically between 18 and 24 nucleotides. Abnormal expression of miRNAs affects drug resistance in tumor cells, and plays an important regulatory role in promoting/inhibiting cancer development [7, 9–11]. For example, Tian et al. [12] found that high levels of miR-106a in the serum of NSCLC patients are significantly reduced after chemotherapy, implying that its high expression could be linked to the growth of NSCLC. Deng et al. [13] discovered a significant downregulation of miR-324-3p expression in A549/DDP cells. Further investigations revealed that miR-324-3p reversed DDP resistance in lung adenocarcinoma A549 cells by inducing ferroptosis mediated by GPX4. Recently, Rao et al. [14] found that miR-219-5p level in DDP-resistant NSCLC cells is lower than that in corresponding parental cells (A549 and SPC-A1), and that miR-219-5p may reverse NSCLC cell resistance to DDP by targeting FGF9. However, free miR-219-5p is unstable and easily degraded by nucleases in blood, as well as exhibiting low transfection efficiency and other issues [15, 16]. Therefore, there is an urgent need for an efficient nanodrug delivery system to achieve effective delivery of miRNAs. Liposomes (Lipo) are drug delivery systems that can meet many requirements for drug formulation therapy with the characteristics of reducing toxic side effects, improving efficacy, and changing pharmacokinetic behavior in vivo [17–19]. Cationic liposomes are the most promising gene delivery carriers and have made significant breakthroughs in cancer treatment [20–22]. For example, Luo et al. [23] prepared a novel cationic liposome DOTAP for loading TNF-related apoptosis-inducing ligand plasmids and achieved promising anti-tumor effects. Jiang et al. [24] prepared cationic liposomes pVAX for miR-143 delivery and found that they can inhibit A549 cell growth and metastasis by targeting CD44v3. However, utilizing cationic liposomes as nanodrug carriers inevitably heightens their immunogenicity and cell toxicity in vivo, thus impeding rapid clearance by the reticuloendothelial system. To address this issue, studies have developed folate (FA)-modified liposomes containing docetaxel, which can not only reduce the immunogenicity of the drug but also achieve precise delivery to the tumor site [25]. We believed that the surface modification of cationic liposomes with FA could effectively improve the immunogenicity and targeting of nanomedicines.

**Scheme 1:**
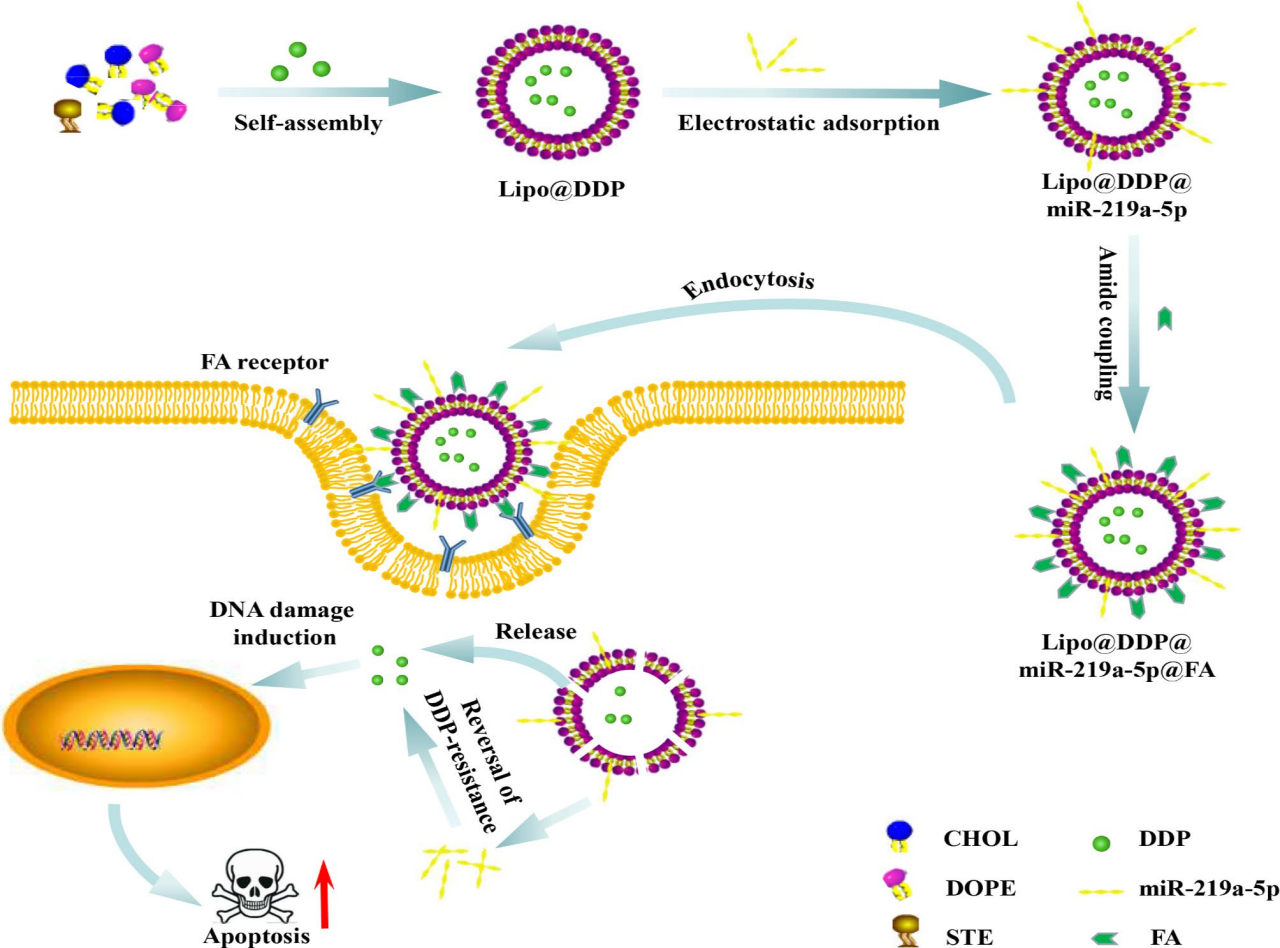
FA-modified Lipo nanomaterials co-deliver DDP and miR-219a-5p to NSCLC cells, effectively inducing apoptosis

To improve the therapeutic landscape of DDP-resistant lung cancer, we developed a FA-modified liposome nanodrug for targeted delivery of DDP and miR-219a-5p (Scheme 1). We initially prepared and characterized Lipo@DDP@miR-219a-5p@FA nanodrug. Following successful formulation, we assessed the stability, safety, cellular uptake efficiency, and cytotoxicity of the nanodrug. Experimental results demonstrated that miR-219a-5p encapsulated within Lipo@DDP@miR-219a-5p@FA nanoparticles exhibited improved stability in serum, while the presence of FA enhanced the uptake efficiency of the nanoparticles by cancer cells. By reversing DDP resistance in A549/DDP cells, miR-219a-5p significantly enhanced the therapeutic efficacy of DDP. Safety assessment indicated that Lipo@DDP@miR-219a-5p@FA nanodrug exhibited no significant toxicity to normal cells and displayed excellent blood compatibility. In conclusion, this study provided a feasible nano-delivery system for the treatment of DDP-resistant NSCLC patients.

## Methods

### Materials

DDP (Cl_2_H_6_N_2_Pt, 65%), FA (C_19_H_19_N_7_O_6_, ≥ 98%), N-hydroxysuccinimide (C_4_H_5_NO_3_, NHS, 95%), 1,2-Dioleoyl-sn-glycero-3-phosphoethanolamine (C_41_H_78_NO_8_P, DOPE, 98%), 1-Ethyl-3-(3-dimethyllaminopropyl)carbodiimide hydrochloride (EDC·HCl, C_8_H_17_N_3_·HCl, 98%), and cholesterol (C_27_H_46_O, CHOL, 95%) were accessed from Shanghai Aladdin Biochemical Technology Co., Ltd. Stearamide (C_18_H_37_NO, STE, 85%) was obtained from Shanghai Macklin Biochemical Co., Ltd. miR-219a-5p was synthesized by Youkang Biotechnology Co., Ltd. All experimental protocols comply with our hospital regulations.

### Preparation of Lipo@DDP@miR-219a-5p@FA

#### Preparation of Lipo@DDP

The method for preparing Lipo@DDP was modified based on the literature [26]. Specifically, 15 mL chloroform was dissolved with CHOL (5.56 mg), DOPE (44.44 mg), and STE (5 mg). To obtain cationic liposomes, the solution was sonicated at 35 ℃ for 15 min before being transferred to a rotary evaporator and evaporated under reduced pressure at 41 ℃. Subsequently, 5 mL of deionized water suspension of DDP (0.4 mg/mL) was added to liposomes, and the mixture was sonicated for 30 s and rotated for 30 min. The resulting suspension was repeatedly extruded through a polycarbonate membrane to form multilayer vesicles, and Lipo@DDP was prepared and stored at low temperatures.

#### Preparation of Lipo@DDP@miR-219a-5p

When using charged materials for nanoparticle synthesis, the ratio of positive to negative charges can affect the stability and electrostatic properties of the nanoparticles. In nucleic acid lipid nanoparticles, positive charges are typically provided by cationic lipids with ionizable ammonium groups (N), while negative charges are conferred by nucleic acid molecules with abundant phosphate groups (P), and the two can be combined through electrostatic adsorption. Referring to previous studies [27], we calculated the N/P ratio and prepared the nanomaterials in a ratio of N/*P* = 3:1. miR-219a-5p (2.5 nmol) was added to 10 mL of deionized water suspension of Lipo@DDP (2 mg) and mixed at a specific molar ratio at 25 ℃ for a certain time. After the reaction was completed, the product was lyophilized and stored at a low temperature.

#### Preparation of Lipo@DDP@miR-219a-5p@FA

The preparation of Lipo@DDP@miR-219a-5p@FA composite nanomedicine was slightly modified based on the previously reported literature [28]. Specifically, 8 mg FA, 3 mg EDC·HCl, and 3 mg NHS were mixed in 10 mL phosphate-buffered saline (PBS) (pH = 7.4) and sonicated for 20 min. Lipo@DDP@miR-219a-5p (2 mg) was mixed with the above solution, stirred in the dark for 24 h at 25 ℃, and the product was collected, purified with water, and the final product was obtained.

### Characterization

Size distribution and zeta potential of the prepared nanodrugs during each preparation process were assessed using dynamic light scattering (DLS) technology (Nano ZS90, Malvern, UK) in triplicate. The morphology of Lipo@DDP@miR-219a-5p@FA was examined using transmission electron microscopy (TEM, Tecnai G20, FEI, Hillsboro, USA) with an accelerating voltage of 200 kV. The structure of each composite nanodrug was determined using Fourier transform infrared spectrometer (FT-IR, Nicolet 5700, USA). Before sample loading, a small amount of each sample was ground with potassium bromide and pressed into a pellet (approximately 1 mm). The wave number range was 4000 − 500 cm^− 1^. The release of DDP in the nanodrug was determined using an inductively coupled plasma optical emission spectrometer (ICP-OES, ICP-5000, Thermo Fisher Scientific, USA).

### Stability of miR-219a-5p in Lipo@DDP@miR-219a-5p@FA

To determine the stability of Lipo@DDP@miR-219a-5p@FA nanodrugs in serum, agarose gel electrophoresis experiments were conducted. In brief, free miR-219a-5p, Lipo@DDP@miR-219a-5p, and Lipo@DDP@miR-219a-5p@FA were separately incubated in PBS solution containing 10% fetal bovine serum (FBS) (v/v) at 37 ℃ for 12 h. Samples were collected at various time intervals (0, 1, 3, 6, and 12 h), and 1.5% agarose gel with 0.5 µg/mL GelRED was used for gel electrophoresis in Tris-acetate-EDTA (TAE) buffer (80 V) for 20 min. The gel was then imaged by a ChemiDocTM imaging system (Bio-Rad, CA, USA) to evaluate the stability of miR-219a-5p in the nanodrug.

### Encapsulation efficiency

To determine the encapsulation efficiency of the liposomal nanomaterial for DDP, we employed a disruption method to completely release DDP from the material. After multiple washes with deionized water, Lipo@DDP@miR-219a-5p@FA was treated with a strong surfactant, Triton, to dissolve the liposomes and release DDP completely. The resulting solution was collected, diluted fivefold with deionized water, and analyzed using high-performance liquid chromatography (HPLC) to determine the Pt content in the mixture, thereby quantifying the amount of DDP. The encapsulation efficiency (EN%) was calculated using the following formula:

$${\rm{EN}}\% \, = \,\left( {1\, - \,{\rm{Cf}}/{\rm{Ct}}} \right)\, \times \,100\% $$

Where Cf represents the amount of free drug, and Ct represents the total amount of the drug in the nanocarrier or liposome suspension.

### In vitro drug release behavior of nanoparticles

The release kinetics of DDP in various nanodrugs under different conditions were determined. To investigate the release behavior of DDP in nanodrugs, 2 mg of free DDP, Lipo@DDP, and Lipo@DDP@miR-219c-5p@FA were separately dispersed in 5 mL of PBS (pH = 7.4), sealed in dialysis bags (MWCO = 3500 Da), and placed in 20 mL of PBS at the same pH on a shaker for dialysis (37 ℃, 100 rpm). The dialysate was collected at each predetermined time point and replenished with an equal amount of PBS. The concentration of DDP in the dialysate was determined by ICP-OES and change in the release rate over time was plotted. In addition, the effect of PBS with pH values of 5.0 and 6.5 on the release behavior of DDP in Lipo@DDP@miR-219a-5p@FA was determined using the same procedure.

### Cell culture

Human lung adenocarcinoma cell line A549 (BNCC100441) and human normal lung epithelial cell line BEAS-2B (BNCC254518) were provided by BeNa Culture Collection (China). A549/DDP-resistant cell line (ATCC 0023) was obtained from Shanghai Beinuo Biology Co., Ltd. A549 cells and A549/DDP-resistant cells were cultured in DMEM with 10% FBS, and BEAS-2B cells were cultured in BEBM with 10% FBS (ATCC, USA). The cells mentioned above were kept in a constant temperature incubator at 5% CO_2_ and 37 ℃ for later use.

### In vitro cellular uptake

The in vitro cellular uptake behavior of the nanomedicine was evaluated. Briefly, A549 and BEAS-2B cells were plated into 6-well plates with glass coverslips at the bottom (5 × 10^4^ cells/well) and incubated for 24 h at 37 ℃ and 5% CO_2_. A549 cells were pre-treated with FA (1 mg/mL) for 1 h before adding Lipo@DDP@Cy5.5-miR-219a-5p@FA (1 mg/mL) for another 4 h of incubation. Cells were rinsed with PBS and fixed with 4% paraformaldehyde. DAPI was the reagent for nuclear staining. A small amount of anti-fluorescence quencher was added to the above cells and then the uptake behavior of various nanomedicines was observed using confocal laser scanning microscopy (CLSM, TCS SP8 X, Germany). The uptake amount of miR-219a-5p in A549 cells was determined by flow cytometry for PBS, Cy5.5-miR-219a-5p, Lipo@DDP@Cy5.5-miR-219a-5p, and Lipo@DDP@Cy5.5-miR-219a-5p@FA groups.

### Measurement of miR-219a-5p transfection efficiency

After treating A549 cells with PBS, free miR-219a-5p, Lipo@DDP@miR-219a-5p, or Lipo@DDP@miR-219a-5p@FA, we performed qRT-PCR experiments to validate the intracellular content of miR-219a-5p, which represents the transfection efficiency of miR-219a-5p. Total RNA was extracted from each experimental group using TRIzol (Thermo Fisher Scientific, USA). 1 µg of total RNA was reverse transcribed into cDNA for miRNA expression analysis using stem-loop primers and the Hifair® II 1st Strand cDNA Synthesis Kit (Yeasen, China). The qRT-PCR products were amplified by SYBR green qPCR (Qiagen, Hilden, Germany) and detected using the RT-PCR detection system (iQ5q, Bio-Rad, USA). PCR conditions were 95 ℃ for 3 min, followed by 40 cycles of 95 ℃ for 12 s and 62 ℃ for 40 s. miR-219a-5p expression was calculated using the equation RQ = 2^−ΔΔCt^ (Ct = quantification cycle for detecting fluorescence). All primers used in this experiment were synthesized by Beijing DingGuochangSheng Biotech. Co. Ltd. (China). U6 was the internal reference for miR-219a-5p in this experiment. Each sample was analyzed in triplicate. The relevant primer sequences are listed in Table 1.

**Table 1:** Primers used in qRT-PCR

Target gene	Primer (5’-3’)	Accession No.	Tm(℃)	Products
miR-219a-5p-JH	GCTCAACTGGTGTCGTGGAGTCGGCAATTCAGTTGAGCAGAATTGC	MIMAT0000276	58.62	-
miR-219a-5p	F: GCCGAGTGATTGTCCAAAC		56.93	87 bp
	R: CTCAACTGGTGTCGTGGA		56.51	
U6	F: CGCTTCGGCAGCACATATACTAA	NC_000015.10	61.10	160 bp
	R: TATGGAACGCTTCACGAATTTGC		60.43	

### 10 cell viability assay and effect of drug combination

To evaluate the impact of nanomedicine on the viability of A549 or BEAS-2B cells, we performed a CCK-8 assay. Specifically, A549 cells were plated into 96-well plates (5 × 10^3^ cells/well) for overnight incubation in an incubator (37 ℃, 5% CO_2_). Cells were rinsed with PBS buffer following the removal of the culture medium. Then, the cells were treated with DDP (DDP in different concentrations: 0.25, 0.5, 1, and 2 µg/mL), Lipo@DDP, Lipo@DDP@miR-219a-5p, and Lipo@DDP@miR-219a-5p@FA in PBS buffer for 24 h. After incubation, cells underwent two washes with PBS and were kept in serum-free culture medium mixed with 10% CCK-8 solution for 4 h. Finally, absorbance at 450 nm was assayed with a microplate reader (iMARK, Bio-Rad, USA), and the cell survival rate was calculated.

The impact of varying drug treatments on the viability of DDP-resistant and non-resistant A549 cells was determined. Briefly, A549 and A549/DDP cells were pretreated with PBS, DDP, Lipo@DDP, Lipo@DDP@miR-219a-5p, and Lipo@DDP@miR-219a-5p@FA (2 µg/mL) for 24 h. After repeating the preceding steps, the cell survival rate of A549 cells was finally assessed using a microplate reader (Victor X, PerkinElmer, USA).

IC50 values were calculated using GraphPad Prism7 software. The combination index (CI) was evaluated using the Chou-Talalay method and CompuSyn software to assess the synergistic effect of DDP and miR-219a-5p [29]. The magnitude of the CI value quantitatively determines the strength and nature of the interaction between drugs (CI > 1 indicates antagonism, CI = 1 indicates additivity, 0.7 < CI < 1 indicates slight synergy, 0.3 < CI < 0.7 indicates synergy, and CI < 0.3 indicates strong synergy). The formula for CI calculation is as follows:

$${\rm{CI}}\, = \,\left( {\rm{D}} \right)1/\left( {{\rm{Dx}}} \right)1 + \left( {\rm{D}} \right)2/\left( {{\rm{Dx}}} \right)2$$

where (Dx)1, (Dx)2 = the concentration of the tested substance 1 and the tested substance 2 used in the single treatment that was required to decrease the cell number by 50% and (D)1, (D)2 = the concentration of the tested substance 1 in combination with the concentration of the tested substance 2 that together decreased the cell number by 50%.

### Hemolysis assay

The hemolysis assay was conducted following established methods described in previous studies [30, 31] to evaluate the biocompatibility of the nanomaterials. Blood samples were collected from healthy volunteers, and 5 mL of 0.9% NaCl solution was used to wash the whole blood. After centrifugation at 2500 rpm for 5 min, the supernatant was discarded. Following the wash, 0.9% NaCl solution was added to obtain a 5% red blood cell (RBC) suspension. Lipo@DDP, Lipo@DDP@miR-219a-5p, or Lipo@DDP@miR-219a-5p@FA was mixed with the RBC suspension in a 1:1 ratio. Equal amounts of deionized water and NaCl solution were added to the RBC suspension as positive and negative controls, respectively. All groups were incubated for 4 h at 37 °C. Finally, photographs were taken to record the hemolysis status.

### Colony formation assay

A549/DDP cells were divided into five groups, and 5 × 10^2^ cells were seeded in each culture dish. The cells were then treated with PBS, DDP, Lipo@DDP, Lipo@DDP@miR-219a-5p, or Lipo@DDP@miR-219a-5p@FA and incubated for 72 h. After removing the old culture medium, fresh medium was added, and the cells were further incubated for 5 d. Finally, the cells were fixed with 4% paraformaldehyde and stained with 0.1% crystal violet (Solarbio, China). Photographs were taken to record the results.

### Wound healing assay

For the wound healing assay, when cells in different groups reached 80% confluence, a 200 µL pipette tip was used to create scratches on the cell monolayer. The cell debris was removed using PBS, and cells were cultured in mediums supplemented with 5% FBS. The width of the wound was measured at 0 and 24 h in three random fields using ImageJ software.

### Cell apoptosis assay

Flow cytometry tested the apoptosis of A549/DDP cells in each drug treatment group. Specifically, A549/DDP cells were treated with varying drugs (2 µg/mL) for 24 h and then washed with PBS buffer. Cells were digested with trypsin without EDTA and resuspended in PBS. Cells were incubated with a PBS buffer containing 1.25 µL of annexin V-FITC for 15 min in the dark. After centrifugation at 1000 ×g for 5 min, the pellet was incubated with 10 µL of propidium iodide. The apoptosis was immediately assayed using the FACSCanto™ II flow cytometer (BD Biosciences, CA, US) equipped with CellQuest software.

### Western blot

A549/DDP cells were collected from each drug-treated group. Protein extraction was performed using RIPA lysis buffer (Beyotime, China), and protein concentration was quantified with a bicinchoninic acid protein assay kit (Bio-Rad Laboratories, Hercules, USA). The protein samples were separated by SDS-PAGE and transferred onto PVDF membranes (The PVDF membrane presenting blots with full-length marker is over-sized for the WB transfer chamber. To adjust the PVDF membrane into the WB transfer chamber, we did cut it to the small-sized ones which can indicate 10–70 KDa. Also, it could blot proteins more stable). The membranes were then blocked with 5% non-fat milk for 2 h. Monoclonal rabbit antibodies against Caspase-3 (ab32351, Abcam, UK), Bax (ab32503, Abcam, UK), and GAPDH (ab181602, Abcam, UK) were added separately for incubation overnight at 4 ℃. Following washes with 0.1% TBST, membranes were incubated with horseradish peroxidase-conjugated goat anti-rabbit IgG (Beyotime, China) for 2 h. Protein bands were assessed with an ECL detection kit (Pierce, Rockford, USA).

### Statistical analysis

The obtained data were processed and analyzed on GraphPad Prism 8.0 (La Jolla, CA). Each experiment was repeated three times, and the values were expressed as mean ± SD. The differences between groups were analyzed using *t*-tests, while one-way analysis of variance was used to measure multiple differences. All differences were deemed statistically significant at *p* < 0.05.

## Results

### Preparation and characterization of Lipo@DDP@miR-219a-5p@FA

miRNAs such as miR-219a-5p play an important role in gene therapy for tumor inhibition, but the instability and poor therapeutic efficacy of single miRNA remain significant issues [32]. To obtain a stable, low-toxicity, highly targeted, and superior drug-release nanocarrier system, we prepared the lipid nanoparticles according to a ratio of N/*P* = 3:1 and successfully encapsulated DDP in Lipo using the thin-film evaporation method and adsorbed miR-219a-5p on the surface of Lipo through the principle of electrostatic adsorption. Subsequently, we coupled FA, which has targeting functionality, to the surface of Lipo@DDP@miR-219a-5p using a coupling reaction to obtain the final product, Lipo@DDP@miR-219a-5p@FA.

**Fig. 1:**
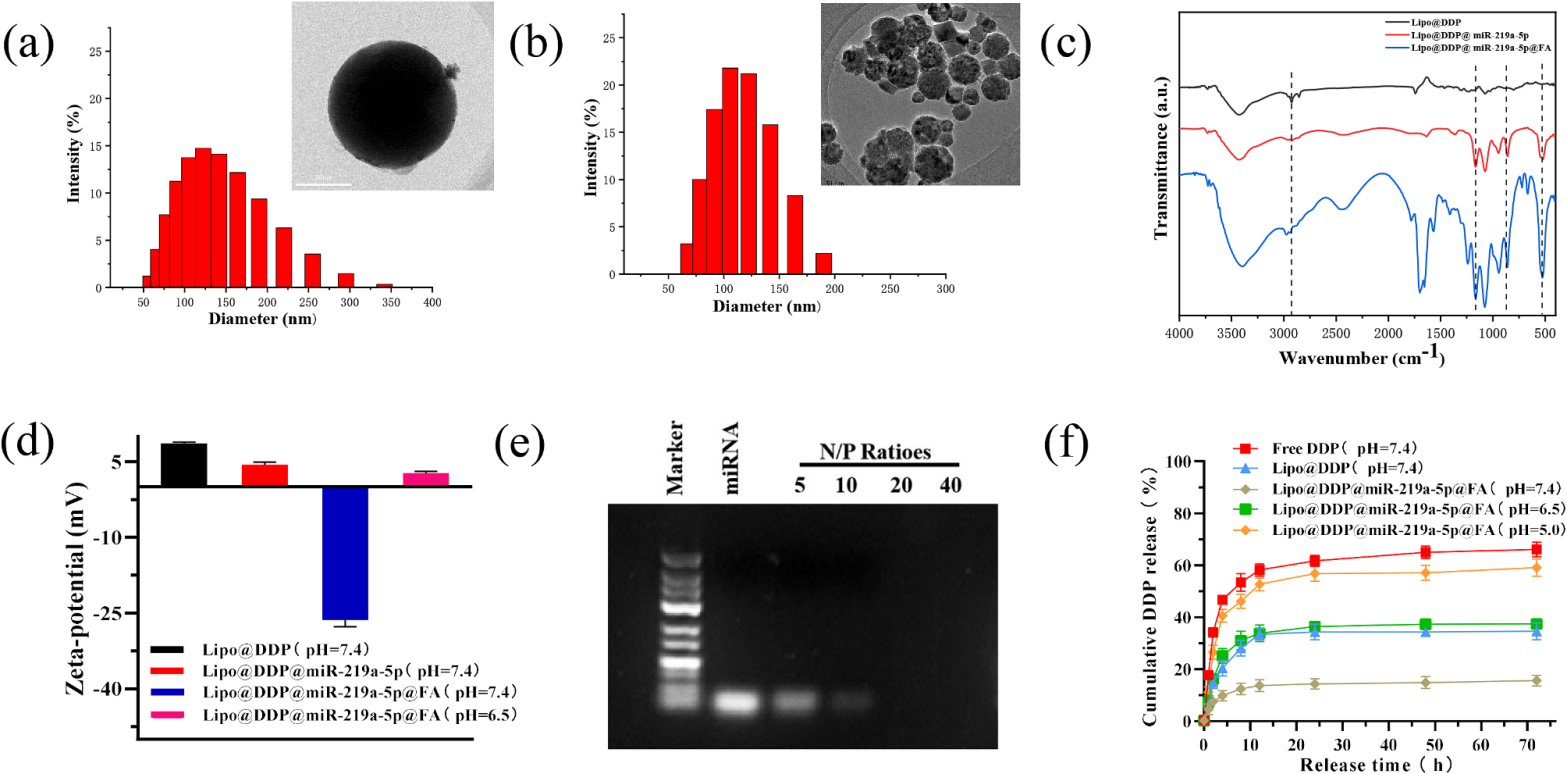
Characterization of nanoparticles and drug release. (**a**, **b**) Particle size distribution and TEM images of Lipo@DDP (**a**) and Lipo@DDP@miR-219a-5p@FA (**b**); (**c**) FT-IR spectra of Lipo@DDP, Lipo@DDP@miR-219a-5p, and Lipo@DDP@miR-219a-5p@FA; (**d**) Zeta potentials of the nanoparticles in PBS buffer at pH 7.4 and 6.5; (**e**) Gel electrophoresis of DDP and miR-219a-5p at different molar ratios in the nanoparticles; (**f**) Drug release profiles of DDP from the nanoparticles at different pH conditions (pH = 5.0/6.5/7.4) within 72 h

Figure 1(a) shows the DLS particle size distribution and TEM image of Lipo@DDP. Particle size was mainly distributed around 120.8 nm, and the morphology was uniform and dispersed as nanospheres. Compared to Lipo@DDP, the particle size of Lipo@DDP@miR-219a-5p@FA increased to approximately 140.3 nm with good dispersion (Fig. 1(b). The structures of the various nanodrugs were characterized by FT-IR (Fig. 1(c). The absorption peaks at 3100 − 2800 cm^− 1^ in the Lipo@DDP spectrum were related to the C-H stretching vibration of the phospholipid acyl chain, while the absorption peaks at 2925 and 2854 cm^− 1^ were related to the symmetric and asymmetric stretching vibrations of -CH_2_ [33]. The absorption peak at 530 cm^− 1^ was attributed to the characteristic absorption peak of _νPt−N_ [34]. In the Lipo@DDP@miR-219a-5p spectrum, the absorption peaks at 3423, 1362, and 946 cm^− 1^ were mainly attributed to the stretching vibration absorption of -NH- in the product. Compared to Lipo@DDP and Lipo@DDP@miR-219a-5p, the intensity of the absorption band at 1636 cm^− 1^ in the Lipo@DDP@miR-219a-5p@FA spectrum increased, which was mainly attributed to the stretching vibration of the material structure and the C = O stretching vibration of the pteridine and benzene ring skeleton of FA. The spectral bands at 1164 and 860 cm^− 1^ could be attributed to the nitrogen-containing heterocycles in FA [35]. These results demonstrated the successful preparation of the Lipo@DDP@miR-219a-5p@FA composite nanodrug.

The electrochemical potentials of Lipo@DDP, Lipo@DDP@miR-219a-5p, and Lipo@DDP@miR-219a-5p@FA were measured using a Malvern Zeta potential analyzer to evaluate the biotoxicity of the nanomedicine, as shown in Fig. 1(d). At pH 7.4, the potentials of the nanoparticles were + 8.6 ± 0.3 mV, + 4.4 ± 0.5 mV, and − 26.35 ± 1.35 mV for Lipo@DDP, Lipo@DDP@miR-219a-5p, and Lipo@DDP@miR-219a-5p@FA, respectively. At pH 6.5, the potential of Lipo@DDP@miR-219a-5p@FA changed from − 26.35 ± 1.35 mV to + 2.70 ± 0.41 mV due to the charge reversal caused by the decomposition of FA in the acidic condition. The potential changes suggested that Lipo@DDP@miR-219a-5p@FA could effectively avoid potential biotoxicity by enhancing the nonspecific electrostatic adsorption between positively charged nanoparticles and negatively charged cell membrane surface in the tumor microenvironment, thus enhancing the internalization of nanoparticles by the cells [36]. Gel electrophoresis assessed nanocomplexes of different molar ratios of DDP and nanomedicine (5:1 ∼ 40:1), as shown in Fig. 1(e). miR-219a-5p in nanomedicine was completely blocked when the molar ratio of DDP to nanocarrier was 20:1.

We analyzed the encapsulation efficiency of DDP in the liposomes using HPLC. Based on the calculation using the formula, the encapsulation efficiency of DDP was determined to be 56.8% (Table 2). The release rate of DDP in various nanomedicines under different conditions was simulated using dialysis experiments, as depicted in Fig. 1(f). In comparison to free DDP and Lipo@DDP, Lipo@DDP@miR219a-5p@FA had the lowest release rate of DDP at pH 7.4 (15.60%). This indicated that Lipo@DDP@miR-219a-5p@FA had good stability during blood circulation. Furthermore, the release rate of DDP was examined in the acidic environment of Lipo@DDP@miR-219a-5p@FA nanomedicine. It was found that the maximum release rate of DDP occurred at pH 5.0 (66.12%) due to the rapid degradation of FA and the rupture of liposome membrane in the acidic condition [37]. This suggested that when Lipo@DDP@miR-219a-5p@FA entered the tumor microenvironment, it could achieve pH responsiveness and rapidly release the nanomedicine while reducing biotoxicity, thereby achieving controlled release of the drug.

**Table 2:** Determination of Pt content by high-performance liquid chromatography

Sample number	Element	Dilution factor	Readings	Content(mg/mL)
1–1	Pt	5	0.1762	0.881
1–2	Pt	5	0.1714	0.857
1–3	Pt	5	0.1708	0.854
			average	0.864

### Stability of miR-219a-5p in serum

**Fig. 2:**
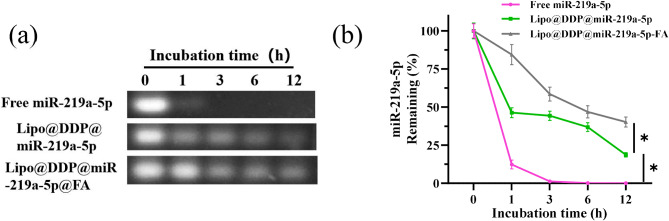
Stability of miR-219a-5p in serum (**a**) Agarose gel electrophoresis of free miR-219a-5p, Lipo@DDP@miR-219a-5p, and Lipo@DDP@miR-219a-5p@FA in fetal bovine serum; (**b**) The retention rate of miR-219a-5p in different nanoparticle formulations. *p < 0.05

The stability of miR-219a-5p encapsulated in the nanoparticle was further investigated using agarose gel electrophoresis (Fig. 2(a, b)). Compared to free miR-219a-5p and Lipo@DDP@miR-219a-5p, Lipo@DDP@miR-219a-5p wrapped by FA degraded less over a period of 12 h (Fig. 2(a)). After incubation in FBS for 12 h, the retention rate of miR-219a-5p in the free miR-219a-5p group was nearly 0%, while in Lipo@DDP@miR-219a-5p and Lipo@DDP@miR-219a-5p@FA groups, the retention rates of miR-219a-5p were approximately 18.64% and 40.20%, respectively (Fig. 2(b)). This further demonstrated that miR-219a-5p encapsulated in the Lipo@DDP@miR-219a-5p@FA nanoparticle was more stable in serum.

### In vitro cellular uptake

**Fig. 3:**
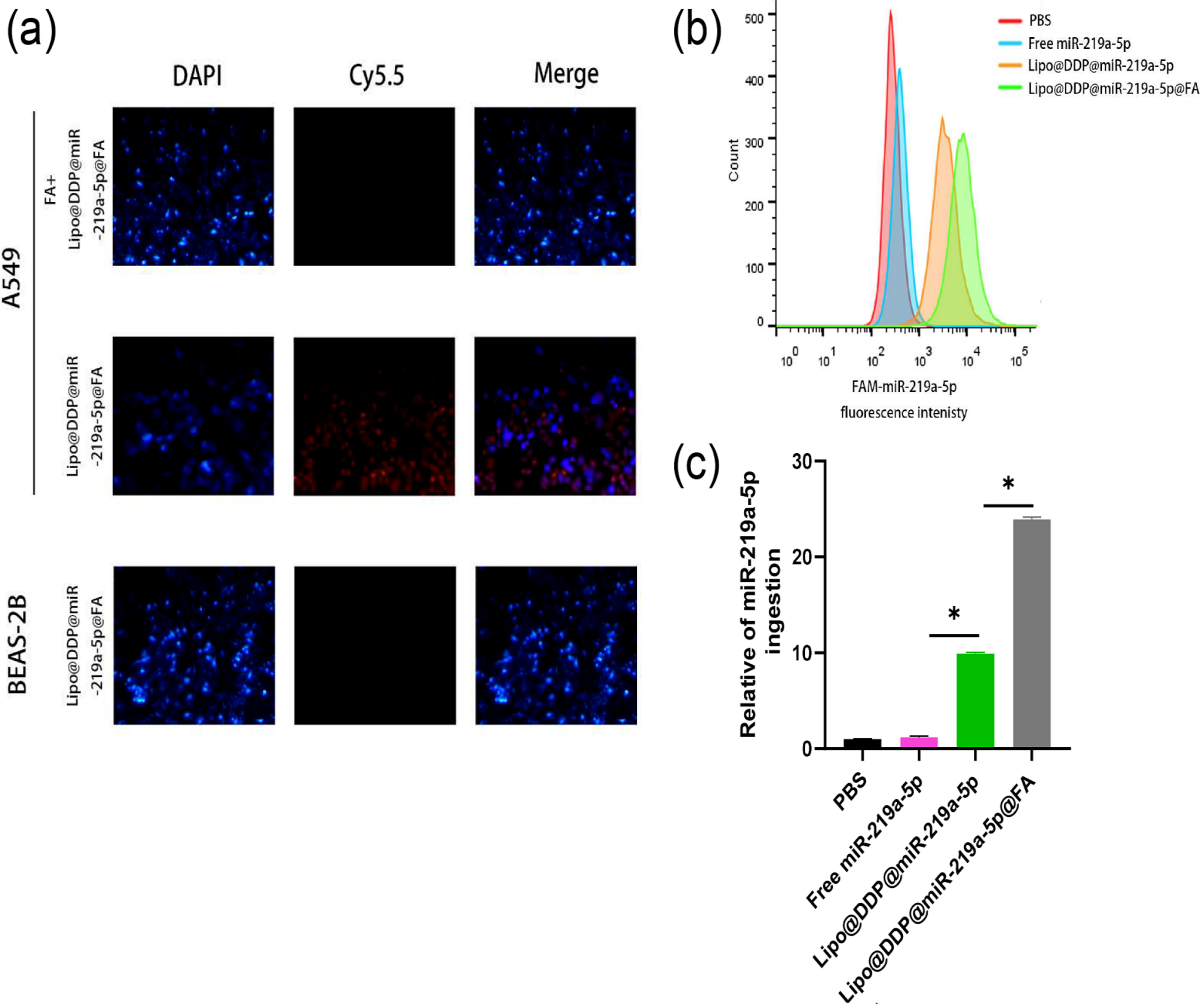
Fluorescence imaging of cellular uptake of nanomedicine and expression levels of miR-219a-5p. (**a**) Uptake behavior of Lipo@DDP@miR-219a-5p-FA in A549 and BEAS-2B cells. Blue: nuclei of cells stained with DAPI; red: miR-219a-5p labeled with Cy5.5; (**b**, **c**) Fluorescence intensity (**b**) and corresponding expression levels (**c**) of miR-219a-5p in A549 cells after co-culturing with different nanoparticles; *p < 0.05

To evaluate the uptake efficiency of Lipo@DDP@miR-219a-5p@FA in cells, we observed fluorescence imaging of the nanoparticles in cells using CLSM (Fig. 3**(a)**). The blue fluorescence represented the nuclei of cells labeled by DAPI, while the red fluorescence represented miR-219a-5p labeled with Cy5.5. It was found that compared with the Lipo@DDP@miR-219a-5p@FA, fluorescence intensity of Cy5.5 in A549 cells pre-treated with FA decreased substantially after co-cultured with Lipo@DDP@miR-219a-5p@FA, indicating that FA pre-treatment reduced the uptake efficiency of DDP in nanoparticles by A549 cells. Compared to BEAS-2B cells, the fluorescence intensity in A549 cells substantially enhanced after treatment with Lipo@DDP@miR-219a-5p@FA, indicating that Lipo@DDP@miR-219a-5p@FA was more easily internalized by A549 cells.

The uptake efficiency of the nanoparticles in cells was further evaluated by flow cytometry (Fig. 3(b)). In comparison to the PBS group, no discernible change was observed in the Cy5.5 fluorescence intensity in the free miR-219a-5p group, indicating that free miR-542-3p was almost unable to internalize into the cells. However, the fluorescence intensity increased significantly after treatment with Lipo@DDP@miR-219a-5p, indicating that encapsulation of miR-219a-5p by Lipo effectively enhanced its internalization. For the Lipo@DDP@miR-219a-5p@FA group, due to the further modification of FA, the uptake efficiency of nanoparticles by cells was significantly improved, and thus FAM-miR-219a-5p showed the highest fluorescence intensity.

To evaluate the transfection efficiency of miR-219a-5p, we extracted total RNA from each experimental group and measured miR-219a-5p levels in A549 cells of different groups using specific primers and qRT-PCR method (Fig. 3((c). Co-culturing with free miR-219a-5p did not increase the expression of miR-219a-5p in the cells. Encapsulation of miR-219a-5p by Lipo@DDP@miR-219a-5p increased miR-219a-5p expression, while Lipo@DDP@miR-219a-5p@FA group notably increased miR-219a-5p level in A549 cells, further demonstrating the level of nanoparticle internalization.

### Safety and biocompatibility of nanocarriers

**Fig. 4:**
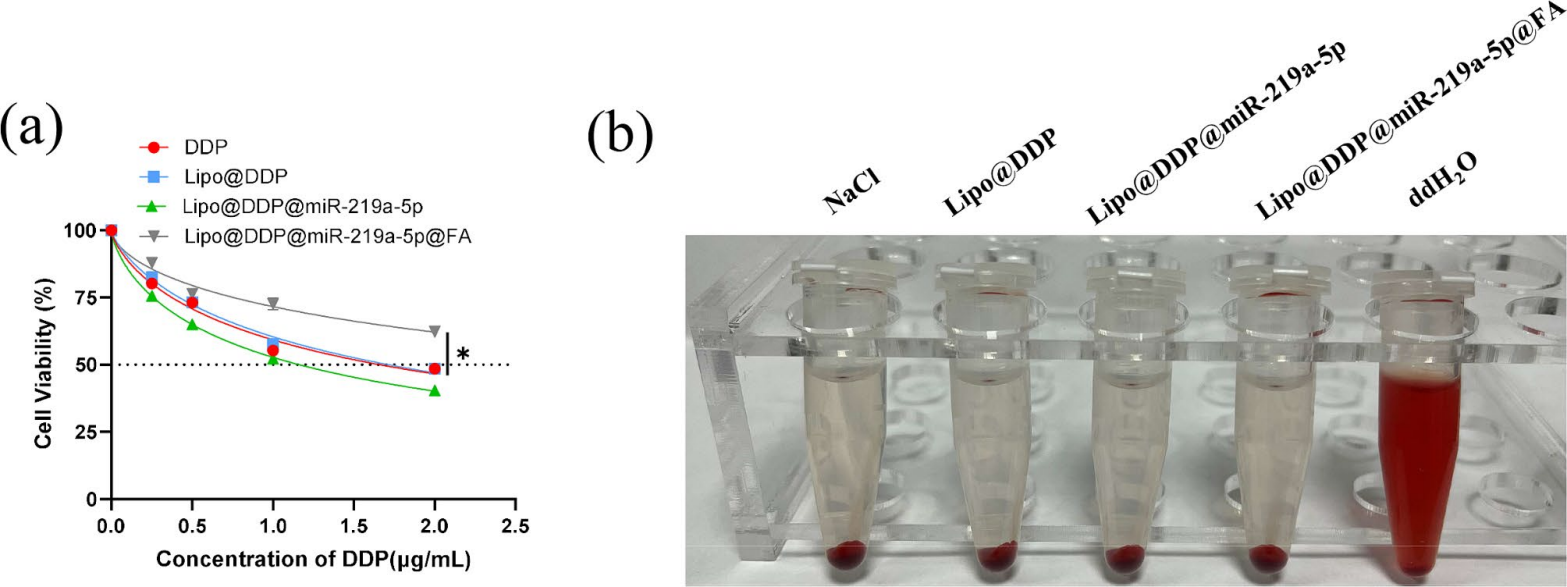
Safety and biocompatibility evaluation of the nanomedicine. (**a**) CCK-8 assay analyzing the effects of free DDP, Lipo@DDP, Lipo@DDP@miR-219a-5p, and Lipo@DDP@miR-219a-5p@FA on the viability of BEAS-2B cells; (**b**) Hemolysis assay examining the blood compatibility of the nanocarriers, with the first and fifth tubes representing negative and positive controls, respectively. *p < 0.05

The safety and biocompatibility of the nanomedicine were further evaluated using CCK-8 assay and hemolysis assay to assess their effects on non-cancerous cells (Fig. 4(a, b)). In Fig. 4(a), compared to free DDP, the Lipo@DDP@miR-219a-5p@FA group exhibited the highest cell viability in BEAS-2B cells, while the cell viabilities in the other groups were similar. This could be attributed to the presence of FA, which prevented the nanocarriers from targeting normal cells and protecting BEAS-2B cells from DDP-induced apoptosis. In the hemolysis assay, the supernatants of the Lipo@DDP, Lipo@DDP@miR-219a-5p, and Lipo@DDP@miR-219a-5p@FA-treated RBCs remained clear (close to the negative control), indicating almost no hemolysis (Fig. 4(b)). These experiments demonstrated that the developed nanotherapeutic in this study exhibited low toxicity to non-cancerous cells and excellent biocompatibility.

### Cell toxicity analysis

**Fig. 5:**
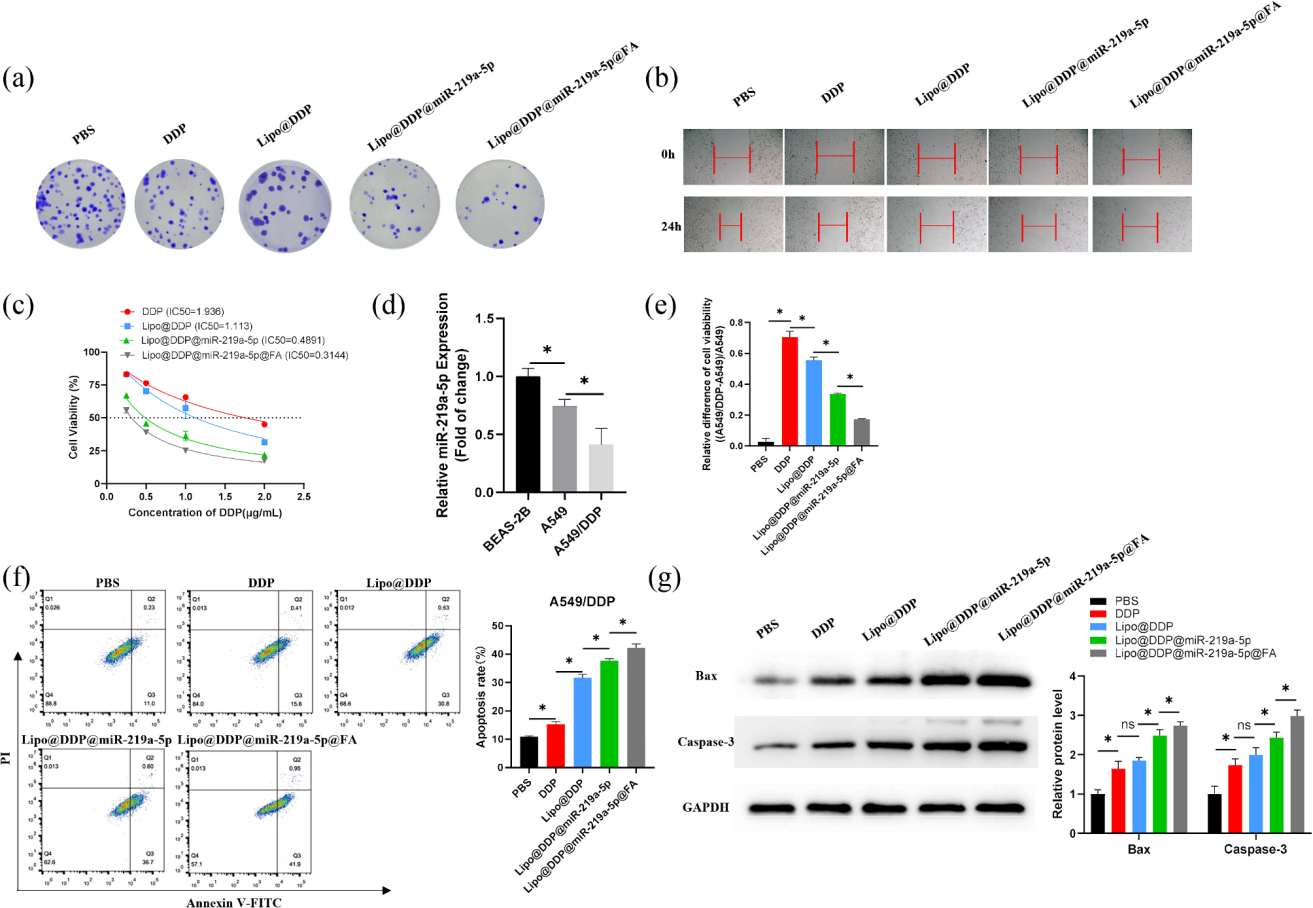
In vitro therapeutic effect of the nanodrugs. (**a**) Colony formation assay results of A549/DDP cells in different experimental groups; (**b**) Cell migration status at 0 and 24 h in different experimental groups using a wound healing assay; (**c**) The influence of various drug treatments on the cell viability of A549 cells; (**d**) The expression levels of miR-219a-5p in BEAS-2B, A549, and DDP-resistant A549 (A549/DDP) cell lines as tested by qRT-PCR; (**e**) The impact of various nanodrug treatments on the cell viability of DDP-resistant/non-resistant A549 cells; (**f**) The results of flow cytometry analysis of the apoptosis of A549/DDP cells in each treatment group; (**g**) The western blot analysis results of A549/DDP cells after nanodrug treatments; *p < 0.05

Cell functional experiments were performed to evaluate the cytotoxic effects of nanomedicine on cancer cells. As shown in Fig. 5(a), Lipo@DDP@miR-219a-5p@FA effectively inhibited the proliferation of A549/DDP cells, and the inhibitory effect was significantly superior to the previous three groups. Co-culturing different drugs with cells and assessing cell migration ability using a wound healing assay were conducted. The results in Fig. 5(b) indicated that at 24 h, the cells treated with PBS, DDP, and Lipo@DDP showed significant migration towards the scratched area, suggesting a strong migratory capacity of cells in these three groups. In contrast, the cells treated with Lipo@DDP@miR-219a-5p and Lipo@DDP@miR-219a-5p@FA exhibited slower migration towards the scratched area, especially in the Lipo@DDP@miR-219a-5p@FA group, where minimal cell migration was observed. This indicated that Lipo@DDP@miR-219a-5p@FA possessed targeting ability and exerted a strong inhibitory effect on cell migration. Furthermore, cell viability and apoptosis were further evaluated in different groups using CCK-8 assay, flow cytometry, and western blot analysis. In Fig. 5(c), IC_50_ values in each treatment group were reduced with the increase in DDP concentration. Under the same DDP concentration, A549 cell viability after treatment with various nanomedicines was ranked as follows: DDP > Lipo@DDP > Lipo@DDP@miR-219-5p > Lipo@DDP@miR-219-5p@FA. The encapsulation of DDP by Lipo enhanced the uptake ability of A549 cells for DDP, resulting in a slight decrease in its cell viability compared to free DDP. The introduction of miR-219-5p in the nanomedicine could enhance the killing effect on A549 cells when combined with DDP. For the Lipo@DDP@miR-219-5p@FA group, due to the modification of FA, it could specifically bind to the FA receptor on the tumor cell surface, increasing the internalization efficiency and thereby better inhibiting the tumor cells.

Downregulation of miR-219a-5p may be related to DDP resistance in NSCLC [14]. Therefore, we studied miR-219a-5p levels in various cell lines (BEAS-2B, A549, and A549/DDP) by qRT-PCR (Fig. 5(d)). miR-219a-5p in A549 cells and A549/DDP cells was markedly downregulated, and its expression was lowest in A549/DDP cells. Subsequently, we dissect the influence of nanomedicine on the cell viability of A549 drug-resistant/non-resistant cells (Fig. 5(e)). Relative differences in cell viability after treatment in each drug group were different from that in the blank control group (PBS group). The biggest difference was in the DDP group, which was DDP > Lipo@DDP > Lipo@DDP@miR-219a-5p > Lipo@DDP@miR-219a-5p@FA. With the addition of miR-219a-5p and FA, the difference between drug-resistant/sensitive A549 cells became smaller and smaller, indicating that miR-219a-5p could effectively reverse the DDP resistance of A549 cells.

Simultaneously, the dose-effect relationship data obtained from the CCK8 assay were used for equipotent comparisons and analysis to provide a quantitative description of the drug combination. A mathematical model was established in the “CompuSyn” software based on the Chou-Talalay method to calculate the CI value, which was found to be 0.83411. Since 0.7 < CI < 1, the prepared nanomedicine exhibited a slight synergistic effect.

Flow cytometry assayed apoptosis of A549/DDP cells following treatment with various drugs (Fig. 5(f)). Each treatment group had a higher apoptosis rate than the PBS group. The apoptosis rate in the Lipo@DDP@miR-219a-5p@FA group was the highest, reaching 41.9%, indicating that the combined administration of miR-219a-5p and DDP, as well as FA targeting modification, could significantly reverse the resistance of A549 cells and enhance the anti-tumor effect. In addition, the western blot showed that in comparison to the PBS group, bands of pro-apoptotic proteins Bax and Caspase-3 were deeper in the DDP and Lipo@DDP groups. For the Lipo@DDP@miR-219a-5p and Lipo@DDP@miR-219a-5p@FA groups, the presence of miR-219a-5p significantly deepened protein bands of Bax and Caspase-3, indicating that miR-219a-5p effectively reversed DDP resistance of A549 cells and played a pivotal role in promoting cell apoptosis (Fig. 5(g)). The above experiments indicated that the Lipo@DDP@miR-219a-5p@FA nanomedicine enhanced the therapeutic efficacy of DDP on A549/DDP cells through the targeting effect of FA and the sensitization effect of miR-219a-5p.

## Discussion

Currently, the combination therapy of platinum-based chemotherapy drugs such as DDP remains the gold standard for first-line treatment of NSCLC. DDP achieves its anti-tumor effect by inhibiting DNA replication and promoting the phosphorylation process of tumor cells. However, long-term treatment with DDP can lead to chemotherapy resistance due to decreased sensitivity of tumor cells [7, 8]. In recent years, cancer therapy based on non-coding RNA has shown great potential, particularly in addressing drug resistance issues [38, 39].

miRNA and small interfering RNA (siRNA) can selectively inhibit the expression of cancer-related genes/mRNAs through RNA interference (RNAi), representing outstanding therapeutic tools for targeted therapy and precision medicine [38]. The successful application of miRNA and siRNA therapies relies on safe and effective nano-delivery strategies targeting tumor cells or the tumor microenvironment. In this regard, several promising nano-delivery systems have been developed for systemic administration and improved tumor-targeted delivery while minimizing side effects. For instance, Safaei et al. [40] developed a transmembrane peptide (TAT)-conjugated liposomal nanocarrier for the delivery of trastuzumab and siRNA targeting breast cancer resistance protein (BCRP) to overcome multidrug resistance in breast cancer. Similarly, Saraswat and colleagues developed an oral lipid nanoparticle-based delivery system encapsulating ARV-825 and vemurafenib (VEM) for the treatment of BRAFi-resistant melanoma [41]. They conducted functional studies of VEM resistance in both 2D and 3D tumor spheroids in vitro and investigated the pharmacokinetics and anticancer efficacy in vivo, effectively demonstrating the application value and potential of lipid-based composite drugs in the treatment of resistant tumors. In this study, we focused on the role of RNA molecules in overcoming DDP resistance in tumors. Numerous studies have demonstrated the involvement of miRNA in regulating DDP resistance in cancer [6, 13, 42]. Among them, miR-219a-5p has been reported to play a crucial role in the development of acquired DDP resistance in NSCLC cells by targeting FGF9 and may serve as a therapeutic target for DDP resistance in clinical practice [14]. However, a single miRNA can be unstable in body fluids such as plasma, easily degraded by serum nucleases, and ineffective in treatment [16, 43, 44]. An ideal drug delivery system is needed to protect miRNA from degradation.

Based on this, we first used the thin-film evaporation method to prepare liposomes encapsulating DDP, and then used electrostatic adsorption to successfully modify miR-219a-5p with a negative charge on the surface of Lipo@DDP. To enable the nanodrug delivery system to target and prevent premature leakage of the nanodrug, we successfully modified FA on the surface of Lipo@DDP@miR-219a-5p using the coupling method and finally obtained well-dispersed and uniformly sized (approximately 135.8 nm) Lipo@DDP@miR-219a-5p@FA nanodrugs. Gel electrophoresis experiments confirmed the stability of the nanodrugs in serum, and degradation of miR-219a-5p in Lipo@DDP@miR-219a-5p@FA was lower than that of free miR-219a-5p and Lipo@DDP@miR-219a-5p within 72 h in FBS environment. Furthermore, Zeta potential analysis presented that the nanodrug had a charge of approximately − 26.9 mV at pH = 7.4, while with weakly acidic conditions (pH = 6.5), the charge was reversed (reaching about + 2.68 mV). These results further indicated the good stability of Lipo@DDP@miR-219a-5p@FA in serum and the reversal of charge in a weakly acidic environment, which was beneficial for promoting the internalization of nanoparticles in tumor cells.

The FA receptor is a frequently investigated target for anticancer drug delivery. It has been verified to be overexpressed in numerous solid tumors, including lung cancer [45, 46], breast cancer [47], liver cancer [48], ovarian cancer [49, 50], etc., while expressed at lower levels in normal tissues. Surface modification of nanodrugs with FA successfully binds to FA receptors on the tumor cell surface, thereby promoting drug internalization. We observed through CLSM that compared with normal BEAS-2B cells, the FAM-labeled green fluorescence and Cy5.5-labeled red fluorescence of Lipo@DDP@miR-219a-5p@FA in A549 cells were significantly increased, indicating a significant increase in the uptake efficiency of miR-219a-5p and DDP in nanodrug by the cells. However, the uptake efficiency of nanoparticles in FA-preprocessed A549 cell groups decreased significantly. Flow cytometry experiments further showed that Lipo@DDP@miR-219a-5p@FA had a better internalization efficiency. These data demonstrated that Lipo@DDP@miR-219a-5p@FA was mediated by FA-induced cellular endocytosis. We also conducted an in vitro safety evaluation of the synthesized Lipo@DDP@miR-219a-5p@FA. CCK-8 results indicated that this nanomedicine showed no significant toxicity to normal cells. The hemolysis assay reflected its excellent blood compatibility. Therefore, Lipo@DDP@miR-219a-5p@FA holds promise for drug delivery and anticancer treatment in DDP-resistant patients.

The differential expression of miR-219a-5p in tumor cells has a significant impact on the occurrence and development of cancer. miR-219a-5p is significantly reduced in cancer cells such as osteosarcoma [51], breast cancer [52], and ovarian cancer [53]. We confirmed through qRT-PCR that miR-219a-5p was significantly downregulated in the DDP-resistant A549 cell line (in comparison to BEAS-2B and A549 cell lines), consistent with previous literature. Gene therapy combined with chemotherapy has shown great prospects in cancer treatment. For example, Zhan et al. [44] embedded miRNA21 inhibitors modified with doxorubicin and cholesterol in exosomal vesicles, showing enhanced tumor accumulation and endosomal escape ability, and notably repressed tumor cell growth. In our cell function analysis, we found that Lipo@DDP@miR-219a-5p@FA has a higher cell-killing effect on resistant/non-resistant A549 cells, mainly due to the DDP sensitization effect of miR-219a-5p and the targeting effect of FA. Furthermore, according to the IC_50_ values of DDP-resistant A549 cells treated with nanomedicine, the combined drug delivery of DDP and miR-219a-5p significantly reversed DDP resistance in A549 cells compared to individual delivery.

The cell apoptosis experiment also demonstrated a significant inhibitory effect of Lipo@DDP@miR-219a-5p@FA on DDP-resistant A549 cells. Bax, a well-known apoptosis gene, plays a crucial role in promoting cell apoptosis through mitochondrial stress [54, 55], and participates in the manipulation of Caspase-3 activity. Li et al. [56] investigated the function of miR-1244 in DDP therapy for NSCLC and reported that the overexpression of miR-1244 inhibits viability of A549 cells treated with DDP, promotes Caspase-3 activity, and increases p53 and Bax protein expression. Overexpression of miR-219a-5p can regulate the p53/bcl-2 signaling pathway and cause significant changes in Caspase-3 [57]. To study the impact of varying nanodrugs on the expression of Bax and Caspase-3 proteins in DDP-resistant cells, it was found that the Bax and Caspase-3 protein bands in Lipo@DDP@miR-219a-5p@FA group were significantly increased, mainly due to reversal effect of miR-219a-5p on DDP-resistant cells, which had a significant inhibitory effect on the growth of DDP-resistant NSCLC cells in combination with DDP.

## Conclusion

In conclusion, this study reported on the use of an FA-mediated Lipo@DDP@miR-219a-5p nanodrug delivery system for the treatment of NSCLC. This system could simultaneously deliver chemotherapy drug DDP and anti-cancer gene miR-219a-5p to A549 cells and could significantly reverse the resistance of DDP during treatment. The experimental results showed that Lipo@DDP@miR-219a-5p@FA exhibited good stability in serum. Compared with BEAS-2B cells, A549 cells had overexpressed FA receptors on their surfaces, which could specifically bind to FA in Lipo@DDP@miR-219a-5p@FA drugs, greatly improving the uptake efficiency of A549 cells for nanodrugs. After entering the cells, the activity of A549 cells was significantly inhibited by the nanoparticles. Moreover, due to the presence of miR-219a-5p, the sensitivity of DDP-resistant A549 cells was effectively increased and further promoted the apoptosis of NSCLC cells in combination with DDP. Although our study has provided substantial evidence at the in vitro level for the targeted treatment of cisplatin-resistant lung cancer using Lipo@DDP@miR-219a-5p@FA, further investigation at the in vivo level is warranted. In the future, we plan to conduct studies on pharmacokinetics and anticancer efficacy in vivo. We believe that this multifunctional nanocarrier system holds great promise as a therapeutic approach for reversing DDP resistance behavior in NSCLC.

### Electronic supplementary material

Below is the link to the electronic supplementary material.

Supplementary Material 1

Supplementary Material 2

## Data Availability

All data generated or analyzed during this study are included in this published article.
