# Supplementary material for: Folate-modified liposomes mediate the co-delivery of cisplatin with miR-219a-5p for the targeted treatment of cisplatin-resistant lung cancer
Source: BMC Pulm Med. 2024 Apr 1;24:159. doi: 10.1186/s12890-024-02938-6 (PMC10986081; doi:10.1186/s12890-024-02938-6)
Supplement: Supplementary file 1 — Supplementary Material 1 [file 12890_2024_2938_MOESM1_ESM.docx]

We note that the images of the original blots appear to be still closely cropped. Are you able to provide images showing full length membranes, with membrane edges visible, for this? Were the blots cut prior to hybridization with antibodies? If original images of full-length blots cannot be provided, please include images of all blots as they are, with membrane edges visible, and for all replicates performed in the Supplementary Information file and include an explanation for the absence of images of adequate length where appropriate in the manuscript (i.e. methods and/or figure legends).

Re: Dear Editor

Thanks for your careful consideration on our original images. But sorry to inform you that we are not able to provide the images with the full-length marker. To make our experiment more precise, we cut the PVDF membrane before hybridization to adjust the transmembrane chamber (clip?) (**Figure 1**).


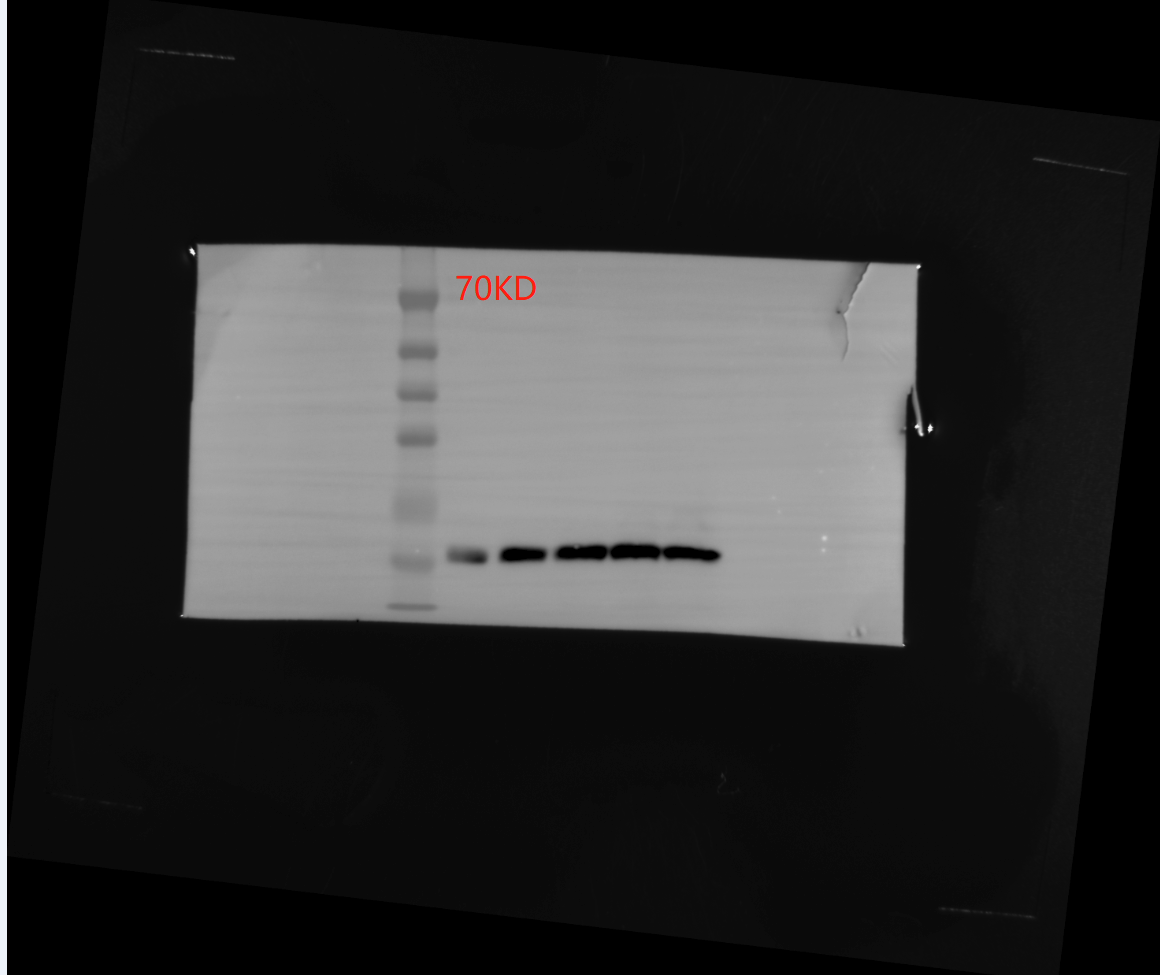


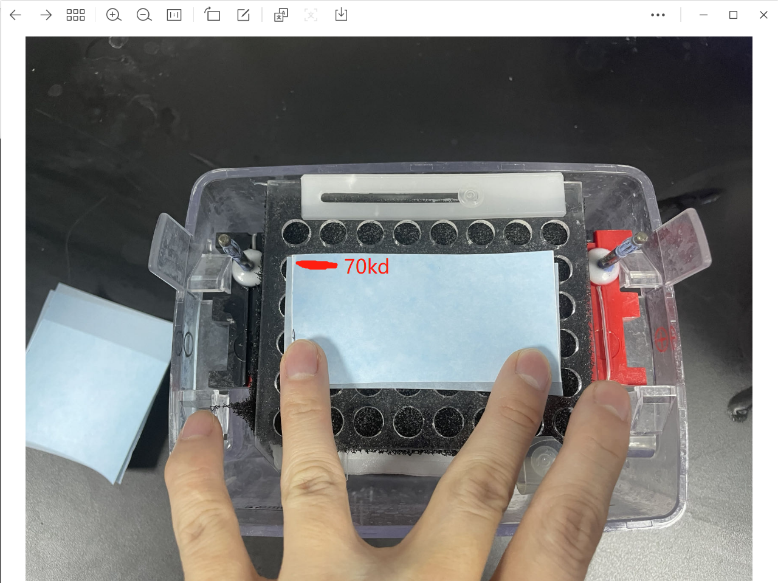


Figure 1 To make the blots more precise and clearer, we use a small size PVDF membrane and put it in the middle of the chamber

If we use big-size PVDF membrane, for one thing we did consider is costing, and another thing which is more important is that the over-sized membrane causes unstable and ugly blots image (Figure 2), due to the limitation of this instrument.


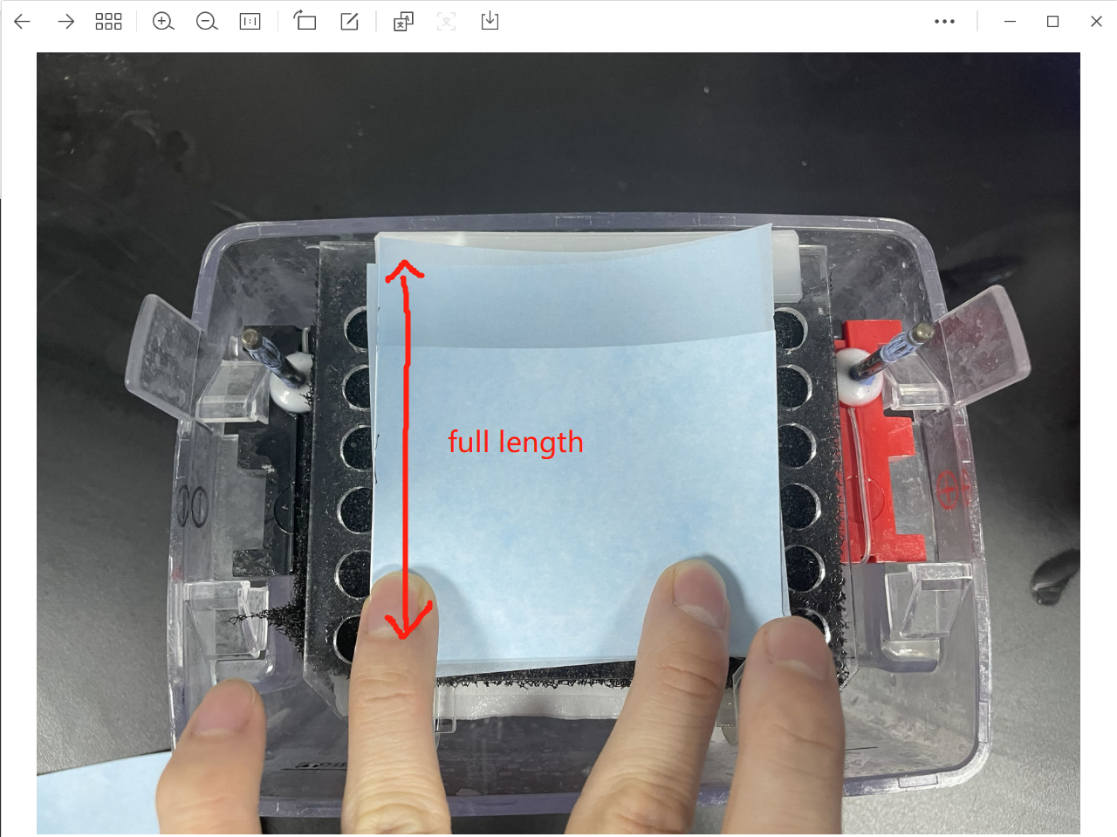


Figure 2 the PVDF membrane is over sized, which could make the unstable blotting.

To fully explain it, we have added a statement in the WB method section in our manuscript. The statement follows below: “ The PVDF membrane presenting blots with full-length marker is over-sized for the WB transfer chamber. To adjust the PVDF membrane into the WB transfer chamber, we did cut it to the small-sized ones which can indicate 10-70 KDa. Also, it could blot proteins more stable”

Thanks for your efforts to make our work more beautiful, and hopefully our explanation could let you know why we process WB in this way!

THANK YOU!
