# Supplementary material for: Folate-modified liposomes mediate the co-delivery of cisplatin with miR-219a-5p for the targeted treatment of cisplatin-resistant lung cancer
Source: BMC Pulm Med. 2024 Apr 1;24:159. doi: 10.1186/s12890-024-02938-6 (PMC10986081; doi:10.1186/s12890-024-02938-6)
Supplement: Supplementary file 2 — Supplementary Material 2 [file 12890_2024_2938_MOESM2_ESM.docx]

Figure1e





Figure2a


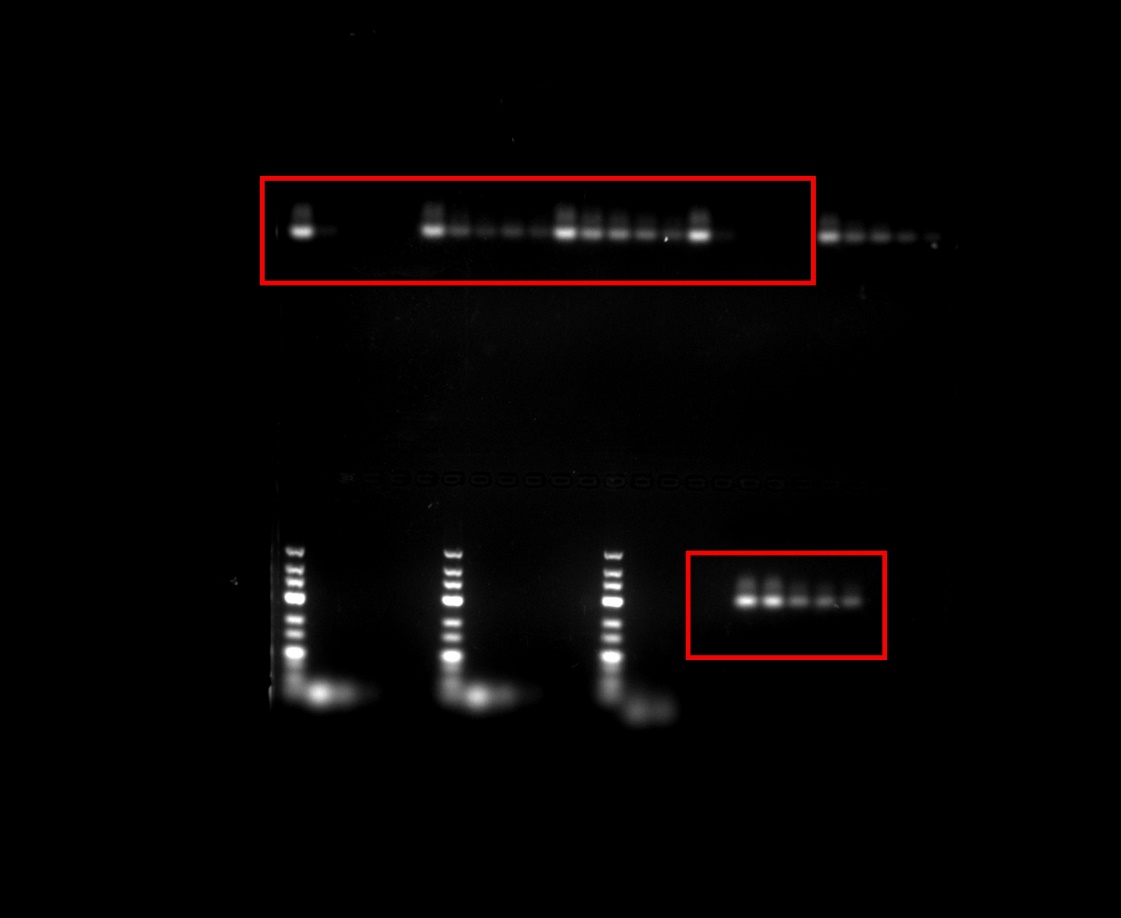


Figure5aPBS


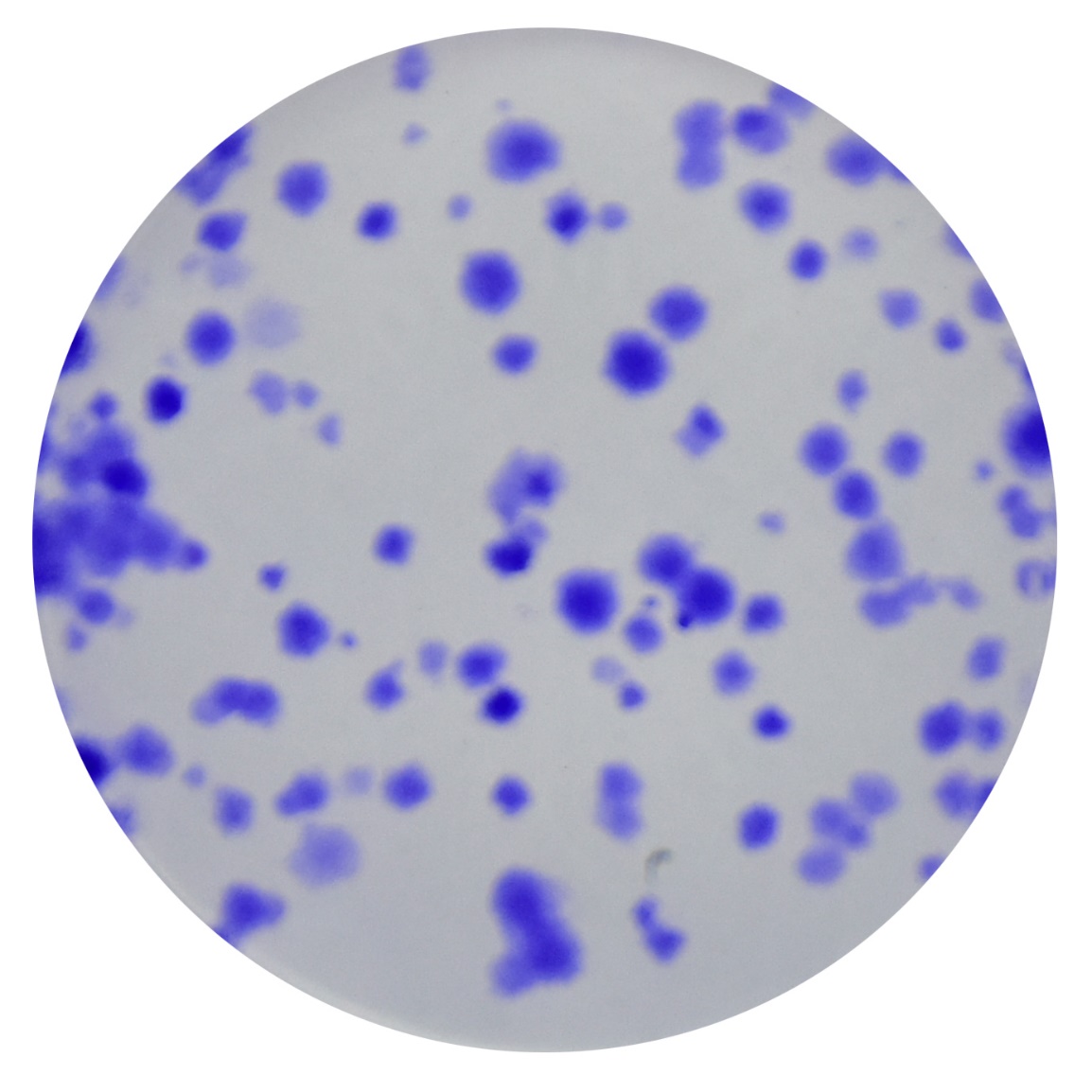


Figure5aDDP


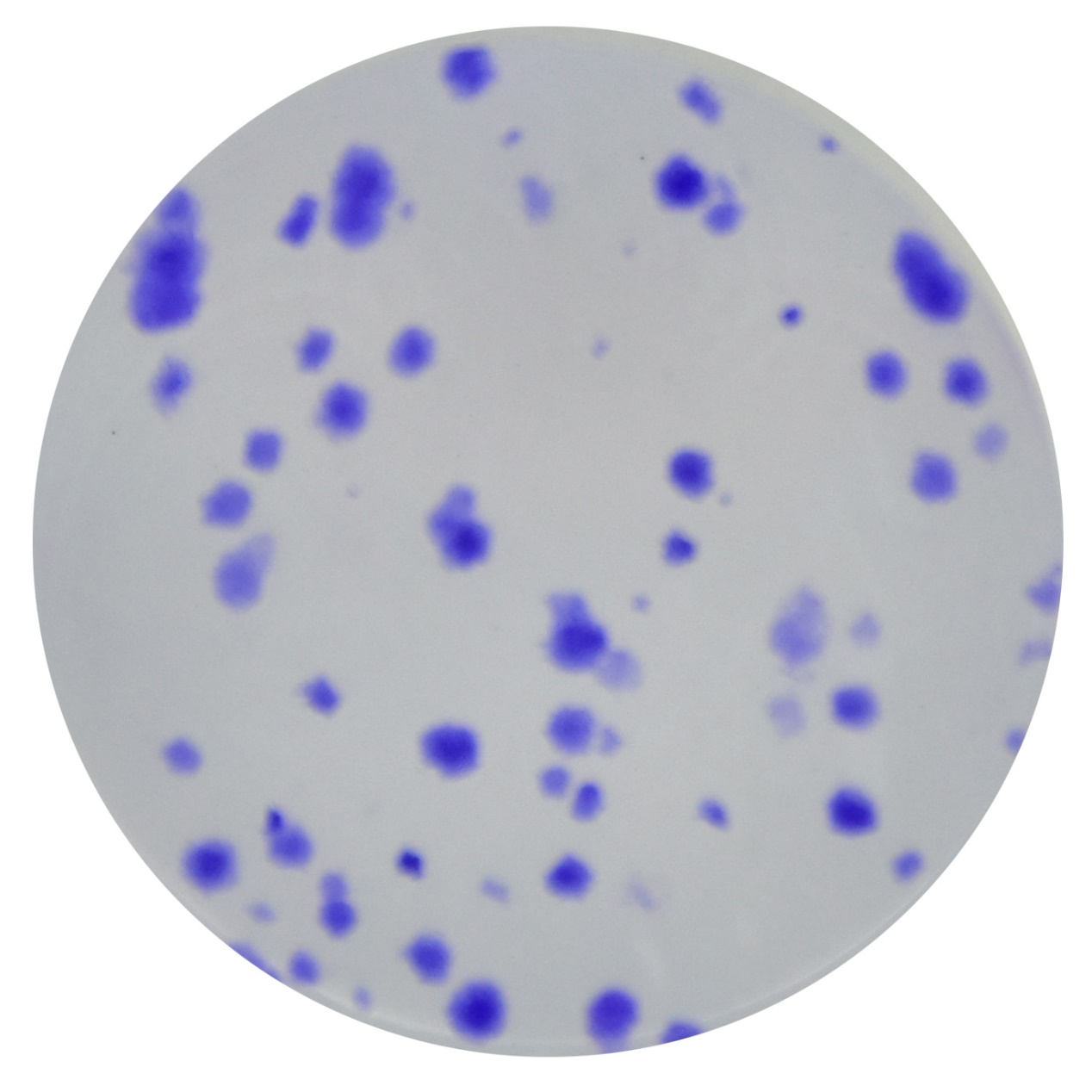


Figure5aLipo@DDP


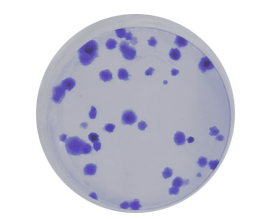


Figure5a Lipo@DDP@miR-219a-5p


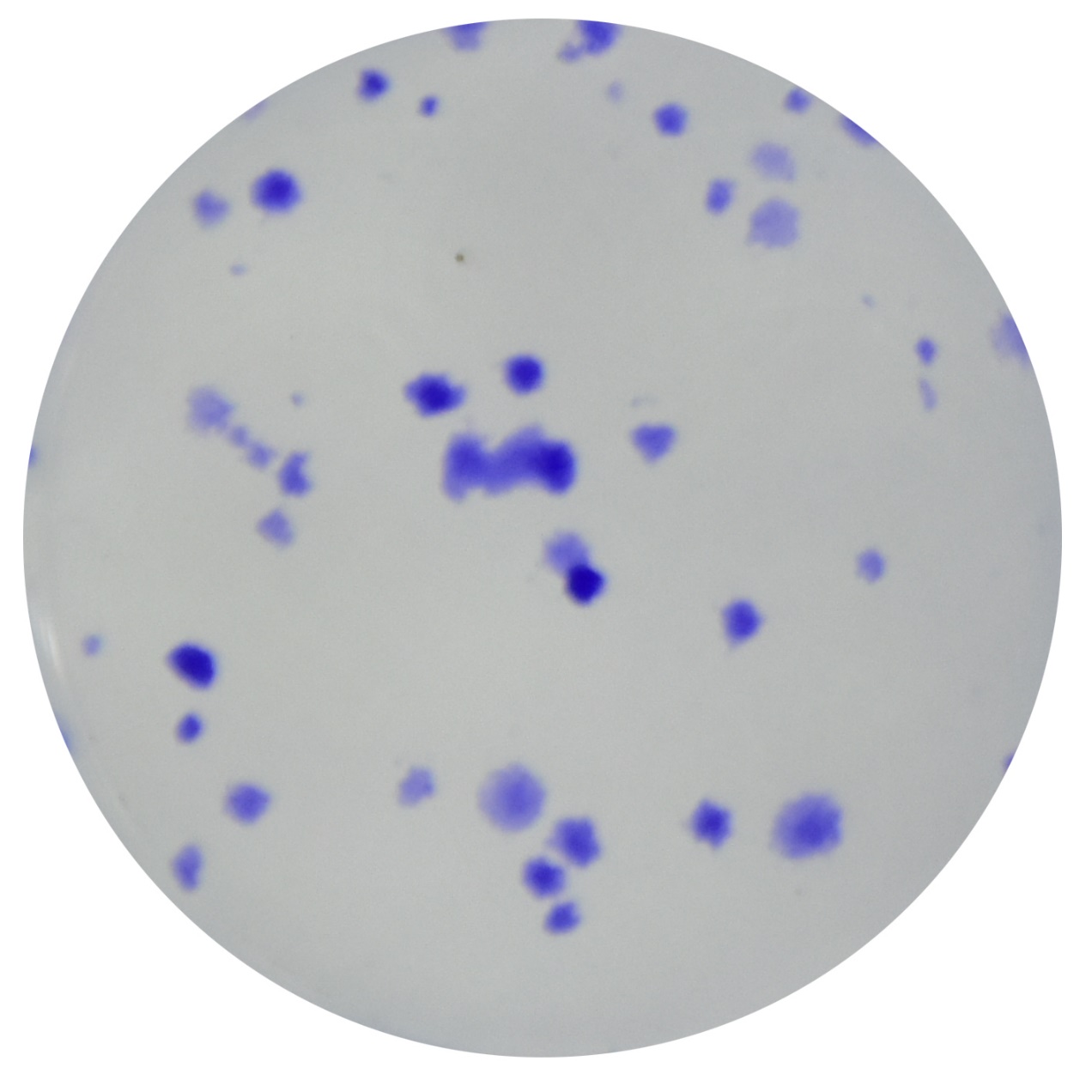


Figure5a Lipo@DDP@miR-219a-5p@FA


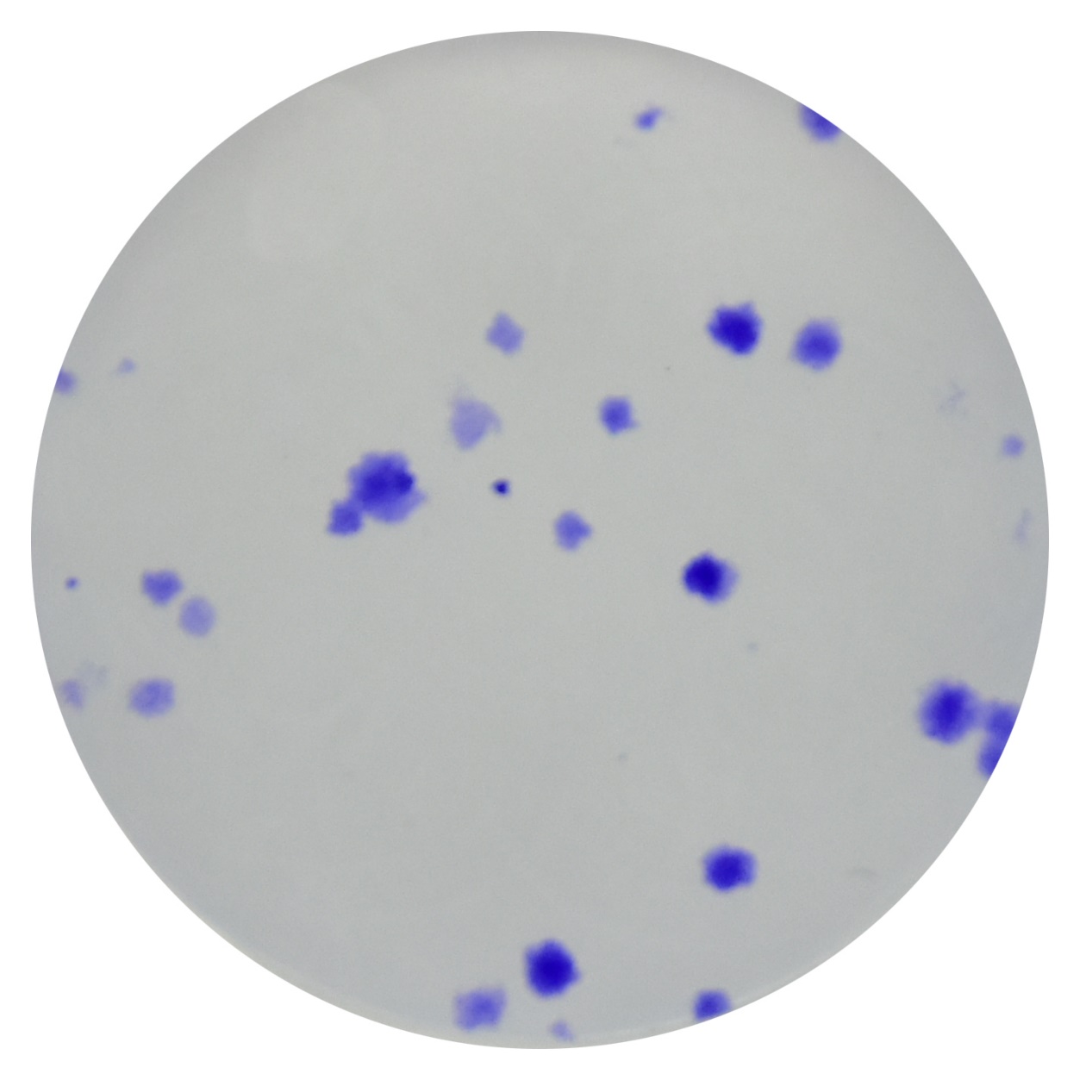


figure5g BAX





figure5g BAX-1





figure5g BAX-1_8bit





figure5g BAX-2_8bit(0)





figure5g BAX-2_8bit(1)





figure5g BAX-2_8bit





figure5g casp-3-1





figure5g casp-3-1_8bit





figure5g casp-3-1_8bit_8bit





figure5g casp-3-2





figure5g casp-3-2_8bit





figure5g casp-3-2_8bit_8bit





figure5g GAPDH A


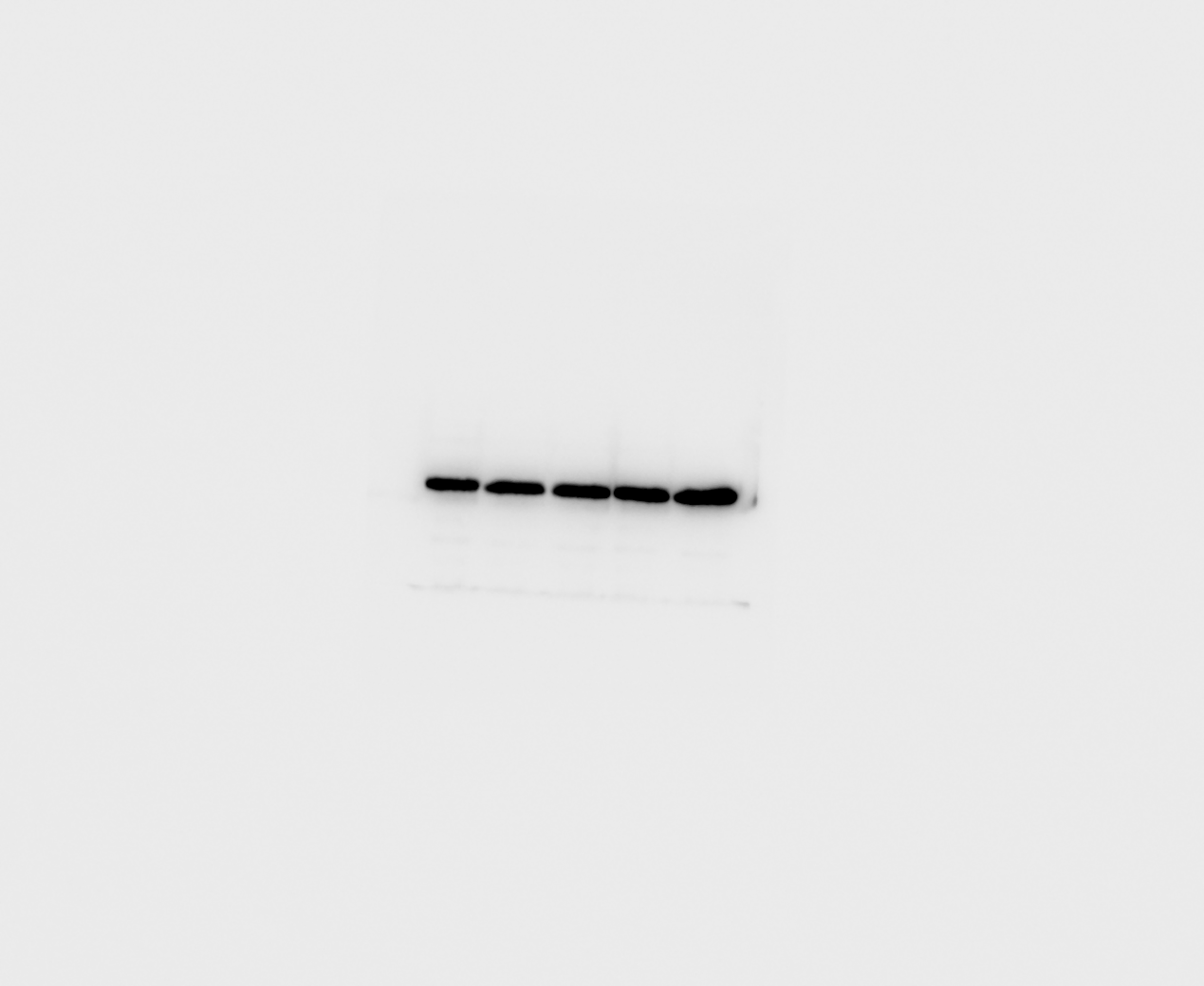


figure5g GAPDH B


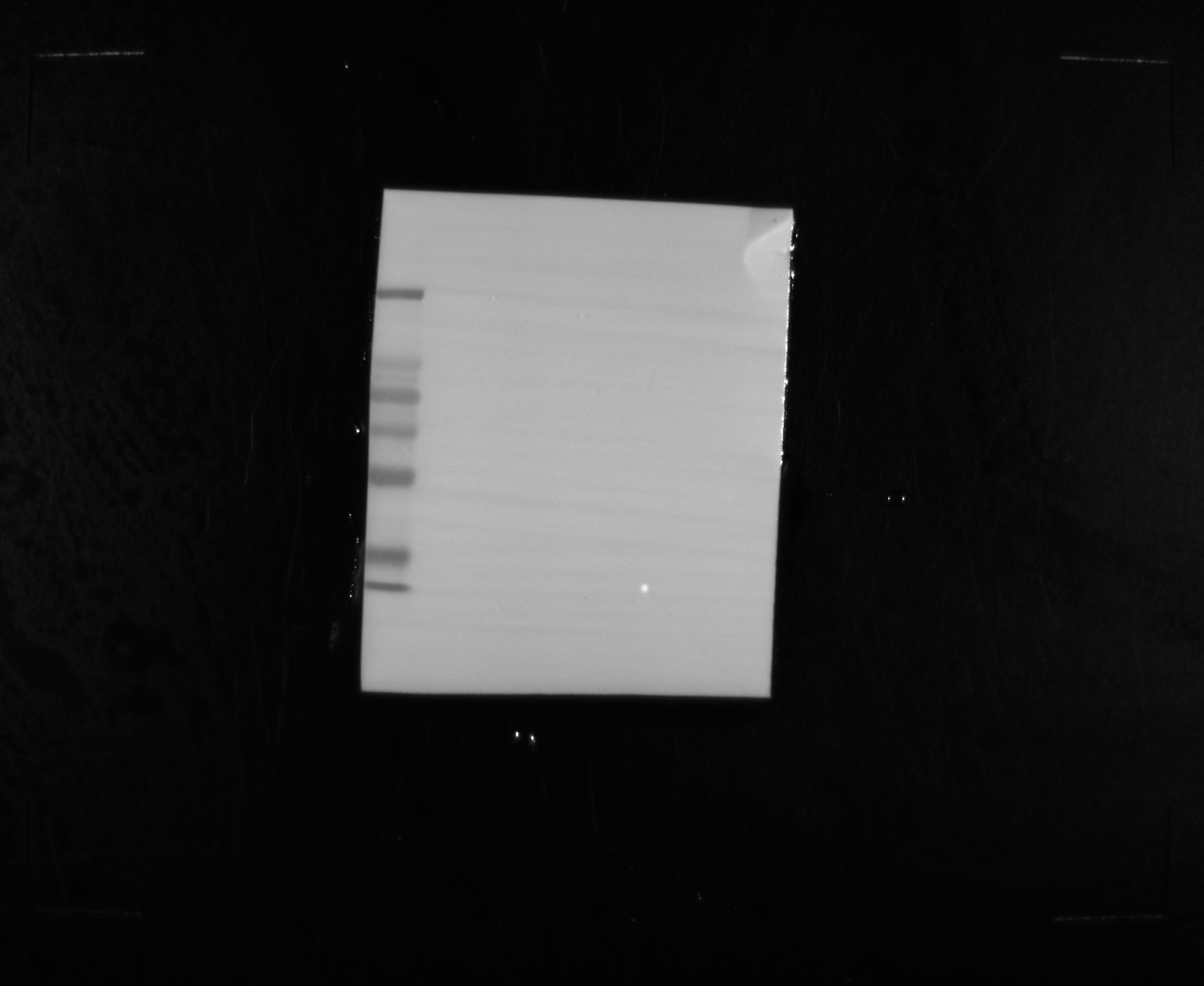


figure5g GAPDH C


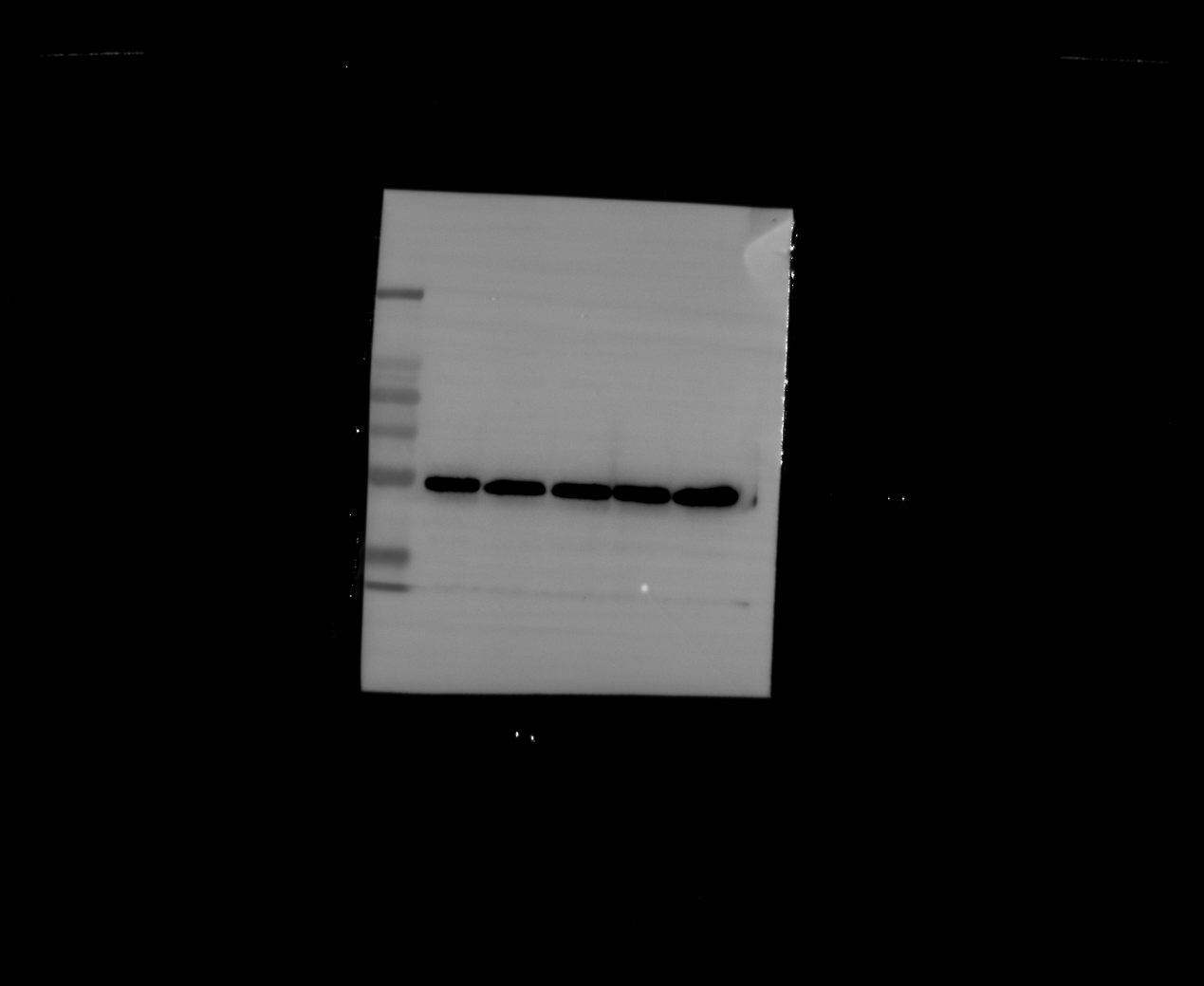


figure5g GAPDH (1)





figure5g GAPDH (2)





figure5g GAPDH (3)
